# Estimating the potential overdiagnosis and overtreatment of acute appendicitis in Thailand using a secondary data analysis of service utilization before, during and after the COVID-19 lockdown policy

**DOI:** 10.1371/journal.pone.0270241

**Published:** 2022-11-03

**Authors:** Jarawee Sukmanee, Rukmanee Butchon, Myka Harun Sarajan, Thanayut Saeraneesopon, Chulathip Boonma, Picharee Karunayawong, Yot Teerawattananon, Wanrudee Isaranuwatchai

**Affiliations:** 1 Health Intervention and Technology Assessment Program (HITAP), Ministry of Public Health, Nonthaburi, Thailand; 2 Saw Swee Hock School of Public Health, National University of Singapore, Singapore, Singapore; 3 Institute of Health Policy, Management and Evaluation, University of Toronto, Toronto, Canada; Oswaldo Cruz Foundation, BRAZIL

## Abstract

**Introduction:**

Acute appendicitis is one of the most common surgical emergencies; however, optimal diagnosis and treatment of acute appendicitis remains challenging. We used the coronavirus disease 2019 (COVID-19) lockdown policy as a natural experiment to explore potential overdiagnosis and overtreatment of acute appendicitis in Thailand. The aim of this study was to estimate the potential overdiagnosis and overtreatment of acute appendicitis in Thailand by examining service utilization before, during, and after the COVID-19 lockdown policy.

**Methods:**

A secondary data analysis of patients admitted with acute appendicitis under the Universal Coverage Scheme (UCS) in Thailand over a 6-year period between 2016 and 2021 was conducted. The trend of acute appendicitis was plotted using a 14-day rolling average of daily cases. Patient characteristics, clinical management, and outcomes were descriptively presented and compared among three study periods, namely pre-pandemic, lockdown, and post-lockdown.

**Results:**

The number of overall acute appendicitis cases decreased from 25,407 during pre-pandemic to 22,006 during lockdown (13.4% reduction) and 21,245 during post-lockdown (16.4% reduction). This reduction was mostly due to a lower incidence of uncomplicated acute appendicitis, whereas cases of generalized peritonitis were scarcely affected by the pandemic. There was an increasing trend towards the usage of diagnostic computerized tomography for acute appendicitis but no significant difference in treatment modalities and complication rates.

**Conclusion:**

The stable rates of generalized peritonitis and complications during the COVID-19 lockdown, despite fewer admissions overall, suggest that there may have been overdiagnosis and overtreatment of acute appendicitis in Thailand. Policy makers could use these findings to improve clinical practice for acute appendicitis in Thailand and support the efficient utilization of surgical services in the future, especially during pandemics.

## Introduction

Acute appendicitis is one of the most common surgical emergencies [1]. Diagnosis of acute appendicitis is based on history and physical, laboratory evaluation, and imaging [2]; however, it can be difficult to diagnose due to similar presentation to other acute abdomen pathologies and the poor predictive value of associated laboratory testing [3]. Rates of negative appendectomy (appendectomy for uninflamed appendix) around 20% have been reported in Lithuania and Israel [4, 5]. Such overdiagnosis and overtreatment not only impact the healthcare budget but also quality of life, as unnecessary appendectomy is associated with higher mortality [6].

The coronavirus disease 2019 (COVID-19) pandemic has affected healthcare systems across the world since the virus was first detected in December 2019 [7]. In Thailand, the peak of the first wave was reached in March 2020, resulting in an announcement of full-scale national lockdown from March 26 to May 3, 2020 [8]. Although these measures were effective in containing the pandemic [9, 10], they also had significant impact on the delivery of health services. For instance, surgical services were affected by a reduced surgical workforce, infection control measures, and elective cancellations [11].

During the COVID-19 pandemic, countries such as China, Egypt and Saudi Arabia observed an overall decrease in total number of patients with acute appendicitis alongside a concurrent increase in complications, such as gangrene or perforation [12–14]. We therefore hypothesise that diagnosis and treatment of cases during the pandemic was restricted to essential procedures only. Thus, we used the lockdown policy as a natural experiment to explore the potential overdiagnosis and overtreatment of acute appendicitis in Thailand. This information can be used to improve clinical practice for acute appendicitis in Thailand as well as to assist in the planning on how to support surgical services during future pandemics. This study aimed to estimate the potential overdiagnosis and overtreatment of acute appendicitis in Thailand by examining service utilization before, during and after the COVID-19 lockdown policy.

## Methods

This study was a secondary data analysis of inpatient data from patients covered under the Universal Coverage Scheme (UCS) in Thailand, comprising approximately 80% of the population. Data were obtained from the National Health Security Office (NHSO) over a 6-year period between 2016 and 2021. Patient-level data were anonymized and de-identified. All patients diagnosed with acute appendicitis were identified using the 10^th^ revision International Classification of Diseases (ICD-10) code K35. Acute appendicitis was classified into acute appendicitis with generalized peritonitis (ICD-10: K35.2), acute appendicitis with localized peritonitis (ICD-10: K35.3), and uncomplicated acute appendicitis (ICD-10: K35.9). The study time frame was divided into three periods: 1) the first period was defined as March-June of 2020 which captured national lockdown in Thailand; 2) for comparison, March-June in 2021 was assigned as a post-lockdown; and 3) March-June in 2019 was used as a pre-pandemic comparison. The changes in the number of patients diagnosed with acute appendicitis among three periods were tested using an interrupted time-series (ITS).

All statistical analyses were performed using R version 4.1.3 (R Foundation for Statistical Computing, Vienna, Austria) [15]. The overall trend of acute appendicitis was plotted from 2016 to 2021 and stratified by age group (children aged under 18 and adults). Rolling averages of daily cases were computed using the ‘rollmean’ function of the zoo package with a 14-day centered rolling window [16]. Descriptive statistics, namely frequency and percentage, were used to explore patients’ characteristics (age, sex, hospital type, health region, and comorbidities), clinical management (diagnostic imaging and treatment modalities), and outcome in the three study periods (pre-pandemic, lockdown, and post-lockdown). Diagnostic imaging included computerized tomography of abdomen (ICD-9-CM: 8801) and ultrasound of abdomen (ICD-9-CM: 8876). Treatment modalities in this study covered open appendectomy (ICD-9-CM: 47.09), laparoscopic appendectomy (ICD-9-CM: 47.01), injection of antibiotics (ICD-9-CM: 99.21), and drainage of appendiceal abscess (ICD-9-CM: 47.2). Patient outcomes were length of stay, cost of hospitalization, and complications (e.g., infection following a procedure (ICD-10: T81.4), in-hospital death, and 30-day readmission). A p-value of 0.05 or lower was considered statistically significant.

The study received approval from the Institute for the Development of Human Research Protections (IHRP) in Thailand (IHRP2020114). Informed consent was not obtained, as the study used the existing national unidentifiable hospitalization data from the NHSO.

## Results

A total of 25,407 (pre-pandemic), 22,006 (lockdown), and 21,245 (post-lockdown) patients were admitted under the UCS with acute appendicitis (Table 1). The number of overall cases decreased by 13.4% during lockdown and 16.4% post-lockdown in comparison to the pre-pandemic period. However, an ITS did not show a significant reduction in the overall number of cases (p = 0.197 lockdown and 0.244 post-lockdown). There was a significant change in hospital type and health region of admission. The proportion of referred patients slightly declined over time, while the prevalence of hypertension, diabetes mellitus, and chronic kidney disease marginally increased.

**Table 1:** Characteristics of patients with acute appendicitis during three study periods.

Characteristics	Pre-pandemic, 2019 (N = 25407)	Lockdown, 2020 (N = 22006)	Post-lockdown, 2021 (N = 21245)	P-value
Age (years), median (IQR)	29 (15,52)	30 (16,53)	30 (16,53)	0.003
Children (<18 years), n (%)	8132 (32.0)	6723 (30.6)	6538 (30.8)	0.001
Male, n (%)	12191 (48.0)	10753 (48.9)	10361 (48.8)	0.105
Hospital type, n (%)				< 0.001
Central hospital	9000 (35.4)	7309 (33.2)	6862 (32.3)	
General hospital	10304 (40.6)	9247 (42.0)	9002 (42.4)	
Community hospital	4046 (15.9)	3769 (17.1)	3798 (17.9)	
Private hospital	475 (1.9)	408 (1.9)	264 (1.2)	
Hospital outside MOPH	1575 (6.2)	1265 (5.7)	1314 (6.2)	
Health service centers and private clinics	7 (0)	8 (0)	5 (0)	
Health region, n (%)				< 0.001
1	3032 (11.9)	2671 (12.1)	2576 (12.1)	
2	1333 (5.2)	997 (4.5)	1113 (5.2)	
3	955 (3.8)	937 (4.3)	892 (4.2)	
4	1485 (5.8)	1226 (5.6)	1247 (5.9)	
5	1743 (6.9)	1547 (7.0)	1402 (6.6)	
6	2216 (8.7)	1786 (8.1)	1599 (7.5)	
7	2222 (8.7)	2053 (9.3)	2092 (9.8)	
8	2418 (9.5)	2200 (10.0)	2136 (10.1)	
9	2932 (11.5)	2456 (11.2)	2434 (11.5)	
10	2159 (8.5)	1853 (8.4)	1763 (8.3)	
11	1808 (7.1)	1637 (7.4)	1515 (7.1)	
12	1703 (6.7)	1459 (6.6)	1416 (6.7)	
13	1394 (5.5)	1175 (5.3)	1051 (4.9)	
14	7 (0)	9 (0)	9 (0)	
Referred patients, n (%)	2266 (8.9)	1866 (8.5)	1734 (8.2)	0.013
Comorbidities, n (%)				
Hypertension	1774 (7.0)	1690 (7.7)	1695 (8.0)	< 0.001
Diabetes mellitus	790 (3.1)	808 (3.7)	747 (3.5)	0.002
Cardiac disease	123 (0.5)	116 (0.5)	104 (0.5)	0.778
Chronic kidney disease	308 (1.2)	334 (1.5)	306 (1.4)	0.012
Diagnosis, n (%)				< 0.001
Acute appendicitis with generalized peritonitis	2424 (9.5)	2213 (10.1)	1989 (9.4)	
Acute appendicitis with localized peritonitis	11597 (45.6)	10728 (48.8)	11096 (52.2)	
Uncomplicated acute appendicitis	11386 (44.8)	9065 (41.2)	8160 (38.4)	

Abbreviation: MOPH, Ministry of Public Health

**Fig 1:**
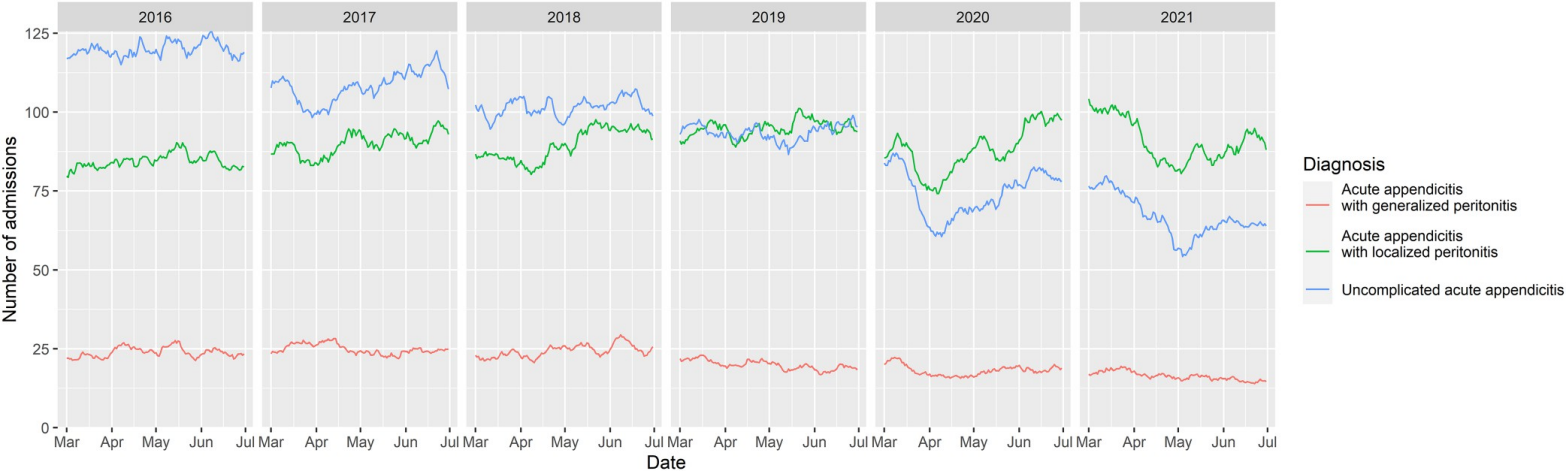
The overall trend of acute appendicitis from 2016 to 2021.

**Fig 2:**
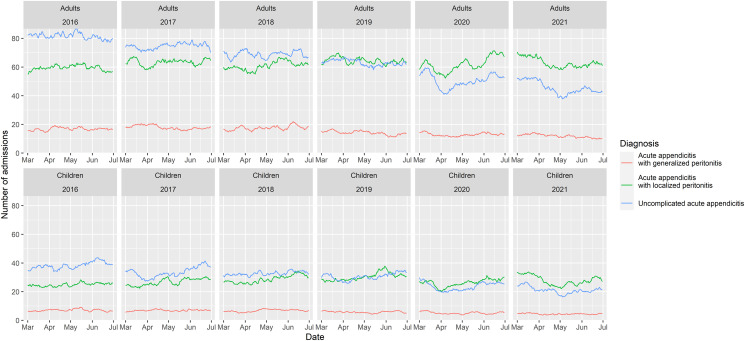
The trend of acute appendicitis from 2016 to 2021, stratified by age group.

Fig 1 shows the overall trend of acute appendicitis from 2016 to 2021. The number of patients with acute appendicitis with localized peritonitis (green line) and uncomplicated acute appendicitis (blue line) significantly decreased during late-March to early-April 2020, then started to climb back in late-April 2020. Another significant drop in the number of acute appendicitis with localized peritonitis and uncomplicated acute appendicitis was observed in early-May 2021. Using an ITS, mean weekly admissions with acute appendicitis with localized peritonitis during the lockdown and post-lockdown periods were significantly decreased (β (95% CI) -141.45 (-242.20, -40.71); p = 0.008 and -212.29 (-379.29, -45.29); p = 0.016), in comparison to the pre-pandemic period. For uncomplicated acute appendicitis, mean weekly admissions reduced by -168.99 (-259.08, -78.90; p < 0.001) and -241.42 (-390.77, -92.08; p = 0.003) during the lockdown and post-lockdown periods, respectively. On the contrary, the trend of acute appendicitis with generalized peritonitis was quite stable. The β coefficients from an ITS model of weekly admissions during the lockdown and post-lockdown periods, when compared with pre-pandemic, were -5.94 (-29.22, 17.33; p = 0.619) and -19.65 (-58.24, 18.94; p = 0.323), respectively. In the age-stratified analysis, similar trends were observed in adults; however, no significant decrease in number of cases was observed among children (Fig 2).

Clinical management and outcomes for patients with acute appendicitis during the three study periods are presented in Table 2. There was an increasing trend towards the use of computerized tomography during the pandemic from 7.4% pre-pandemic to 9.9% during lockdown, and 12.6% post-lockdown, whereas the usage of ultrasonography remained steady (~4%). During the three study periods, open appendectomy was the most performed treatment modality (~88%). The proportions of open appendectomy, laparoscopic appendectomy, and antibiotics were small (< 1%) and remained unchanged over time. The median of length of stay was 3 days (interquartile range; IQR = 2 to 4 days) across the three study periods. The median cost of hospital stay paid by NHSO marginally increased from 10,682 Thai Baht (THB) (IQR = 8,675 to 11,386 THB) during the pre-pandemic period to 11,112 THB (IQR = 9,095 to 12,260 THB) during lockdown, and 12,196 THB (IQR = 9,861 to 14,295 THB) in the post-lockdown period. The rates of in-hospital mortality, infection following a procedure, and 30-day readmission over the three study periods were approximately 0.7%, 0.2%, and 7.0%, respectively.

**Table 2:** Clinical management and patient outcomes for acute appendicitis during the three study periods.

	Pre-pandemic, 2019 (N = 25407)	Lockdown, 2020 (N = 22006)	Post-lockdown, 2021 (N = 21245)	P-value
Diagnostic imaging, n (%)				
Computerized tomography	1870 (7.4)	2180 (9.9)	2686 (12.6)	< 0.001
Ultrasonography	984 (3.9)	880 (4.0)	897 (4.2)	0.157
Treatment modalities, n (%)				
Open appendectomy	22223 (87.5)	19416 (88.2)	18642 (87.7)	0.039
Laparoscopic appendectomy	256 (1.0)	243 (1.1)	257 (1.2)	0.114
Antibiotics	60 (0.2)	41 (0.2)	29 (0.1)	0.047
Drainage of appendiceal abscess	57 (0.2)	64 (0.3)	56 (0.3)	0.356
Length of stay (days), median (IQR)	3 (2,4)	3 (2,4)	3 (2,4)	0.472
Hospitalization costs paid by NHSO (THB), median (IQR)	10682.2 (8675.2,11385.8)	11112.2 (9094.6,12260.5)	12195.7 (9861.0,14295.4)	< 0.001
Complications, n (%)				
Infection following a procedure	145 (0.6)	170 (0.8)	140 (0.7)	0.026
In-hospital death	66 (0.3)	43 (0.2)	54 (0.2)	0.297
30-day readmission	1899 (7.5)	1508 (6.8)	1500 (7.1)	0.027

Abbreviation: NHSO, National Health Security Office

## Discussion

In this study, we observed a significant decrease in the number of uncomplicated acute appendicitis cases during the COVID-19 lockdown. On the other hand, rates of acute appendicitis with generalized peritonitis and acute appendicitis in children were not affected by the pandemic. We further observed an increasing trend in the hospitalization costs paid by NHSO and use of diagnostic computerized tomography for acute appendicitis, but no significant change in treatment modality, during the COVID-19 pandemic.

During the COVID-19 lockdown, there was a decrease in the number of admissions due to acute appendicitis without generalized peritonitis, which aligns with the findings of previous studies. The reduction in other studies varied from 12.9% to 40.7% depending on study settings (single hospital, multiple centers, or population-based) and country (China, Croatia, Germany, Israel, and United States) [12, 17–20]. Enforcement of curfew law between 22:00 to 04:00 on 2 April 2020 along with travel restriction [8] could result in a reduction in the number of admitted patients with acute appendicitis during March-June of 2020 in Thailand. In April 2021, the emergence of the alpha variant has led to a rebound in COVID-19 cases in Thailand [21]. These additional variants might increase or at least maintain patient’s fear of getting COVID-19 infection from a hospital and accelerate a previously downward trend, which led to even lower number of acute appendicitis cases without generalized peritonitis than in 2020.

Although additional measures to visit hospitals during lockdown and fear of being infected with COVID-19 from a hospital visit may have led to a delay in diagnosis and more severe presentation, we did not find any significant increase in the rate of acute appendicitis with generalized peritonitis or complications either during or after the lockdown period. This finding may reflect the effort to maintain effective communication between the Thai government and the public, as well as continued access to health services, even during the lockdown [10]. A similar retrospective, multicenter cohort study in the United States showed similar findings of a decreased incidence of uncomplicated appendicitis and no change in complicated appendicitis [22]. On the other hand, studies from China, Nepal, and the United States reported a higher incidence of acute complex appendicitis (e.g., suppurative, perforated, or gangrenous appendix) [13, 20, 23], which could be due to differences in settings and healthcare systems.

With the finding of stable complication rates overtime despite a decrease in hospital admissions for acute appendicitis, we conclude that there may be potential overdiagnosis and overtreatment for acute appendicitis in Thailand. By subtracting the number of cases in 2020 (an ideal scenario) from those in 2019 and multiplying by the median cost of hospital stay for acute appendicitis paid for by NHSO in 2020, we estimate the medical care costs to the UCS of potential overdiagnosis of acute appendicitis to be approximately 60 million THB annually. We hypothesise that overdiagnosis and unnecessary treatment may result from the pressure on higher level hospitals (central and general hospitals) to perform surgery for patients referred with suspected acute appendicitis. Surgeons at higher level hospitals may risk being sued if they miss a case of acute appendicitis cases; hence, opting for patient observation either at home or in hospital may not be worth the risk. The hospital experiences no financial loss from overdiagnosis and overtreatment under the UCS scheme, as inpatient services are reimbursed based on case-mix classifying patient conditions into groups according to resources consumed [24].

An increase in hospitalization cost over the 3 periods were observed to be approximately 500 to 1,000 THB (equivalent to 10–30 US dollars) and this increase in average costs might be explained by the inflation as an increase in hospitalization cost over time has also been observed in our previous work [25]. Moreover, the NHSO allowed reimbursement for additional costs of personal protective equipment (PPE) at the amount of 740 THB per set in patients with positive COVID-19 test or pending result [26] since all patients undergoing surgery during the early pandemic were recommended to take the COVID-19 test before surgery [27].

To the best of our knowledge, this is the first study to explore potential overdiagnosis and overtreatment of acute appendicitis in Southeast Asia (Thailand) using COVID-19 lockdown as a natural experiment. Given that potential overdiagnosis and overtreatment not only impact the healthcare budget but can also lead to adverse health outcomes from unnecessary surgery, our results can be used to improve clinical practice for acute appendicitis in Thailand in the future including as a case study for other settings with similar context. Nevertheless, there are some limitations in data availability in this study as we used claims data from hospitals. The UCS database accounts for only approximately 80% of the Thai population and does not cover the private sector. We used only data available from existing administrative database, which did not include clinical information. For example, there was no information regarding pathological confirmation of diagnosis and the number of cases might have been overestimated. Additionally, diagnoses of acute appendicitis with or without perforation or rupture, or with peritoneal abscess, were recorded using the same ICD-10 code version 2010 (K35.3). Thus, we could not explore the trends of each diagnosis, which have different degrees of severity. Finally, only descriptive analysis was performed in this study; therefore, risk factors for overdiagnosis and overtreatment and other clinical information should be explored in future research.

This study demonstrated a significant reduction in the number of admissions with uncomplicated acute appendicitis during and after the COVID-19 lockdown in Thailand, whereas the number with more severe generalized peritonitis and complications remained stable over time. These findings suggest that there was potential overdiagnosis and overtreatment of acute appendicitis cases in Thailand before the pandemic. With a better understanding of emergency surgical service utilization during the COVID-19 pandemic, policy makers could improve clinical practice for acute appendicitis in Thailand and optimize the utilization of surgical services in future.

## Supporting information

S1 File Aggregated dataset on number of appendicitis cases per day used for plotting Fig 1. (CSV)

S2 File Aggregated dataset on number of appendicitis cases per day used for plotting Fig 2. (CSV)
